# Can 3-dimensional cranial ultrasound be used to successfully reconstruct a 2-dimensional image without compromising on image quality in a neonatal population?

**DOI:** 10.1007/s00247-024-05886-9

**Published:** 2024-02-27

**Authors:** Rachel M. Roberts, João Alves Rosa, Siân Curtis, Adam P. R. Smith-Collins, Martin Kidd, Savvas Andronikou

**Affiliations:** 1 Department of Neuroradiology, West Wing Level 1, John Radcliffe Hospital, Headley Way, Headington, Oxford, OX3 9DU UK; 2 Neuroradiology Department, Southmead Hospital, North Bristol NHS Trust, Bristol, UK; 3 Department of Medical Physics & Bioengineering, St Michael’s Hospital, Southwell Street, Bristol, UK; 4 Regional Neonatal Intensive Care Unit, St Michael’s Hospital, Southwell Street, Bristol, UK; 5 Neonatal Neuroscience, University of Bristol Medical School, Southwell Street, Bristol, UK; 6 Centre for Statistical Consultation, Department of Statistics and Actuarial Sciences, University of Stellenbosch, Stellenbosch, South Africa; 7 Department of Radiology, Children’s Hospital of Philadelphia, Philadelphia, PA USA; 8 Perelman School of Medicine, University of Pennsylvania, Philadelphia, PA USA

**Keywords:** Brain, Imaging, three-dimensional, Intensive care units, neonatal, Neonate, Ultrasonography

## Abstract

**Background:**

Cranial ultrasound is frequently performed in neonatal intensive care units and acquiring 2-dimensional (D) images requires significant training. Three-D ultrasound images can be acquired semi-automatically.

**Objective:**

This proof-of-concept study aimed to demonstrate that 3-D study image quality compares well with 2-D. If this is successful, 3-D images could be acquired in remote areas and read remotely by experts.

**Materials and methods:**

This was a prospective study of 20 neonates, who underwent both routine 2-D and 3-D cranial ultrasounds. Images were reconstructed into standard views extracted from the 3-D volume and evaluated by three radiologists blinded to the acquisition method. The radiologists assessed for the presence of anatomical landmarks and overall image quality.

**Results:**

More anatomical structures were identified in the 3-D studies (*P*<0.01). There was a trend that 3-D ultrasound demonstrated better image quality in the coronal plane, and 2-D in the sagittal plane, only reaching statistical significance for two coronal views and two sagittal views.

**Conclusion:**

Overall, this study has demonstrated that 3-D cranial ultrasound performs similarly to 2-D and could be implemented into neonatal practice.

## Introduction

For the last three decades, advances in perinatal care have helped to reduce the morbidity and mortality of severe neurological conditions in the neonatal population, especially in preterm neonates [1]. Advances in cranial ultrasound (US) and magnetic resonance imaging (MRI) have contributed hugely to this, and they remain the most frequently used imaging techniques to assess the brain in the perinatal period [2]. Due to its portability, relative low cost, and absence of requirement for sedation, US remains the primary method used to screen and evaluate intracranial abnormalities in the neonatal intensive care unit (NICU) [3], such as haemorrhage, parenchymal abnormalities, congenital malformations, and hydrocephalus.

Becoming proficient in performing conventional 2-dimensional (D) cranial US can be time-consuming and requires extensive training. Two-D US acquisition remains highly operator-dependent and interpretation of the images acquired by an operator other than the interpreting radiologist has been shown to reduce diagnostic confidence and accuracy [4, 5]. Nevertheless, this secondary review is often required in day-to-day neonatal medical practice. Additionally, the need to obtain diagnostic quality cranial US studies to guide management is a contributing factor for the transfer of fragile neonates to higher levels units, where experienced operators are available.

Three-D US has been shown to produce diagnostic images in multiple radiology subspecialities, ranging from breast to obstetrics, and inclusive of both the fetal and neonatal brain [6–9, 9, 10]. Irrespective of the method used, 3-D US acquisition is semi-automated, with reduced input from the operator [11]. Additionally, 3-D US allows the acquisition of a volumetric data set of the whole area of interest, reducing the potential to miss relevant anatomical regions, and increasing intra- and inter-individual reproducibility through permitting reconstruction in multiple planes [12]. Shorter acquisition times have also been shown for acquiring 3-D studies [8, 9], which is of clear benefit to sick neonates immobilised by medical equipment. Therefore, 3-D US offers multiple solutions to many of the limitations of 2-D US.

The ability of 3-D US to depict certain intracranial pathologies in the neonatal population has been demonstrated to be similar to 2-D US, with potential advantages such as more accurate volume measurements [8, 9]. However, it is not feasible to compare every possible intracranial pathology and the ability to identify normal anatomical landmarks is a good surrogate, as many pathologies manifest as alterations to these structures.

The aim of this proof-of-concept study was to determine the feasibility of a larger-scale study comparing the ability of 2-D and 3-D cranial US to demonstrate specific predetermined intracranial anatomical landmarks, whilst providing early evidence of the ability of semi-automated 3-D US to produce images of similar diagnostic quality to 2-D US performed by an experienced operator.

## Materials and methods

This was a prospective study conducted at a level 3 NICU, approved by the Health Research Authority (IRAS 237123), and funded by the Royal College of Radiologists of the UK.

### Patients

An initial sample size of 20 neonates was deemed as adequate for this initial proof-of-concept study.

Consecutive neonates admitted to the NICU, requiring a conventional 2-D cranial ultrasound as part of their clinical care during the period of recruitment (March 2019 to February 2020), were included. This included a mix of full-term and pre-term infants, a small number of whom had intracranial pathology. Exclusion criteria were inability to obtain written informed consent from the patient’s parent or guardian, major congenital abnormality, clinical instability, or concern that the added time to acquire the extra scan (3-D) would impact negatively on patient’s care, and previous inclusion in the study.

Although the main objective of this study was to compare visualisation of anatomical landmarks and ability to produce views of diagnostic quality, a small number of patients with intracranial pathology were included to allow assessment of the potential impact of pathology on the performance of 3-D US compared to 2-D and to inform on the design of future studies. Pathology included hydrocephalus, hyperechogenic periventricular white matter, thickened choroid, and choroidal cysts. Once the target number of patients with pathology was reached (five patients), further patients with pathology were excluded.

### Three-dimensional safety

Three-D US has been demonstrated to subject the patients to the same acoustic exposure as conventional 2-D with comparable thermal index and significantly lower mechanical index in obstetric and fetal US [13, 14]. Three-D imaging does not introduce any additional safety considerations [15]. Thermal index and mechanical index were monitored during scanning as per British Medical Ultrasound Society guidelines [16, 17]. Additionally, a dedicated risk assessment was conducted at the host institution and the additional 3-D study was deemed to be of very low risk; this is available upon request.

### Image acquisition

**Fig. 1:**
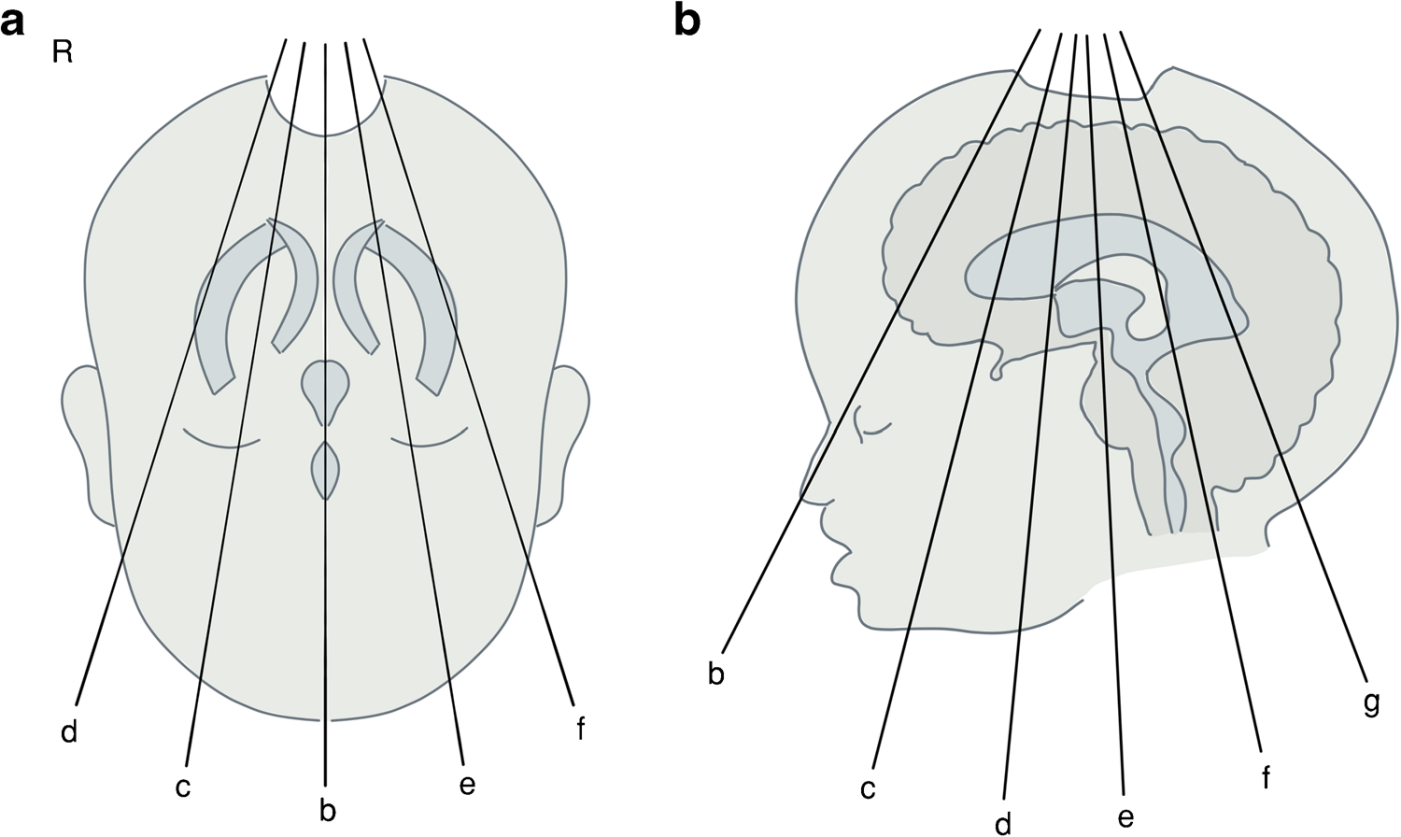
Standard normal 2-dimensional ultrasound probe angulation at the anterior fontanelle for the six coronal (**a**) and five sagittal (**b**) views

**Fig. 2:**
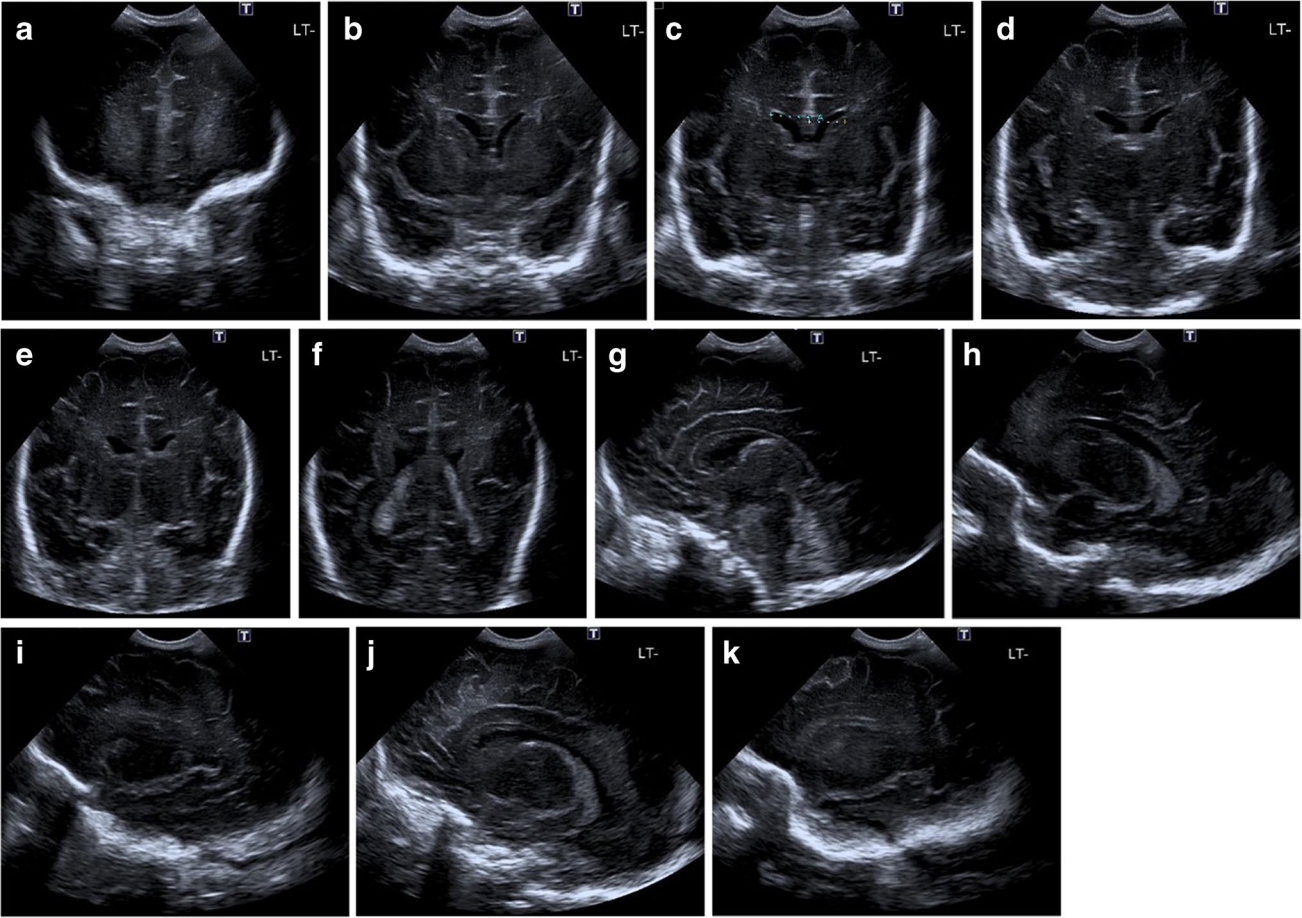
Standard normal 2-dimensional ultrasound views in a 41-day-old boy. Views show the frontal lobe (**a**), frontal horns (**b**), third ventricle and Sylvian with callipers measuring the bicoronal width of the frontal horns of the lateral ventricles (**c**), temporal uncus and Sylvian (**d**), tentorium (**e**), choroid plexus (**f**), midline (**g**), right caudothalamic groove (**h**), right temporal lobe **(i**), left caudothalamic groove (**j**), and left temporal lobe (**k**)

Based on the current literature [18] and current local NICU clinical practice, six coronal and five sagittal views were pre-defined as standard, allowing a total of 77 anatomical landmarks to be analysed per US study (Figs. 1 and 2, Table 1).

**Table 1:** Pre-determined standard coronal and sagittal views and relevant anatomical landmarks for each view

Coronal views	Frontal lobe	Frontal horns	Third ventricle and Sylvian	Temporal uncus and Sylvian	Tentorium	Choroid plexus
Anatomical landmarks	• R frontal lobe • L frontal lobe • R cerebral sulci • L cerebral sulci • Interhemispheric fissure	• R frontal horn • L frontal horn • R cerebral sulci • L cerebral sulci • Interhemispheric fissure	• R frontal horn • L frontal horn • R deep nuclei • L deep nuclei • Third ventricle • R Sylvian fissure • L Sylvian fissure • R temporal lobe • L temporal lobe • R cerebral sulci • L cerebral sulci • Corpus callosum • Cavum septum • Interhemispheric fissure	• R Sylvian fissure • L Sylvian fissure • R temporal uncus • L temporal uncus • Third ventricle • R transverse fissure • L transverse fissure • R cerebral peduncle • L cerebral peduncle • R cerebral sulci • L cerebral sulci • Cerebellum • Interhemispheric fissure	• Echogenic tentorium / cerebellum • R lateral ventricle • L lateral ventricle • R thalamus • L thalamus • R cerebral sulci • L cerebral sulci • Interhemispheric fissure	• R lateral ventricle with choroid • L lateral ventricle with choroid • R periventricular white matter • L periventricular white matter • R cerebral sulci • L cerebral sulci • Interhemispheric fissure
Sagittal views	Midline	Right caudothalamic groove	Right temporal	Left caudothalamic groove	Left temporal	
Anatomical landmarks	• Genu CC • Splenium CC • Cingulate gyrus • Surface sulci • Vermis • Midbrain / pons • Third ventricle	• Caudate half moon • Thalamus full moon • Choroid glomus • Cingulate gyrus • Surface sulci	• Parietal lobe • Frontal lobe • Temporal lobe • Sylvian Sulci	• Caudate half moon • Thalamus full moon • Choroid glomus • Cingulate gyrus • Surface sulci	• Parietal lobe • Frontal lobe • Temporal lobe • Sylvian sulci	

*CC* corpus callosum, *L* left, *R* right

For each patient, a 3-D followed by a 2-D study was sequentially acquired by a single operator (A.S.C.), a consultant neonatologist with over ten years’ experience in cranial US acquisition, interpretation, and teaching.

**Fig. 3:**
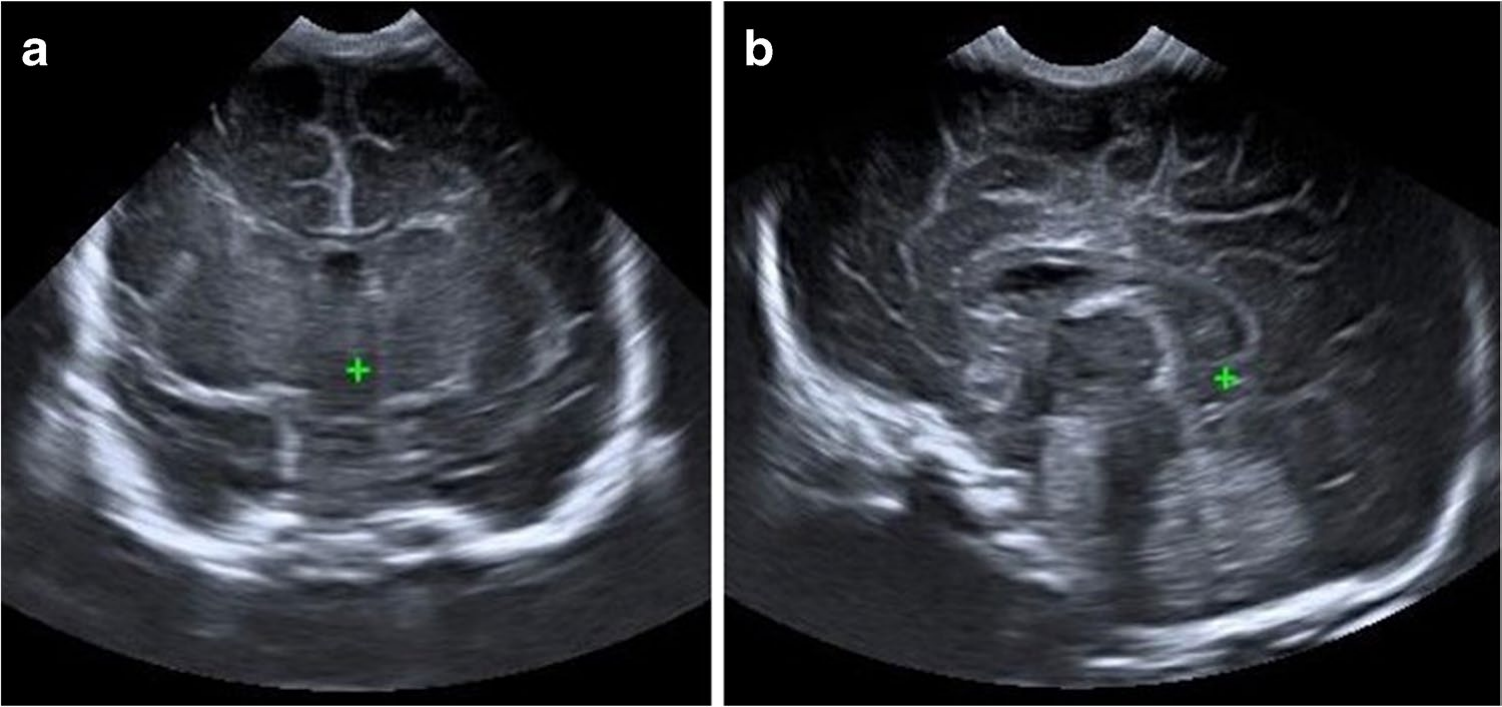
A 41-day-old girl with a normal cranial ultrasound (US). Images obtained in the baseline probe location at the anterior fontanelle for the 3-dimensional US volume acquisition, for the coronal (**a**) and sagittal (**b**) planes

The 3-D study consisted of two volumes acquired consecutively in the coronal and sagittal planes. This was performed using a Toshiba Canon Aplio 500 Ultrasound Machine (Canon Medical Systems, Ōtawara, Japan) and a Toshiba PVT-681MVL (11CV3) 3-D/4-D 3.6–11 MHz probe, a mechanical 3-D/4-D probe, which was chosen for its small footprint, a necessity for scanning neonatal heads. The only operator-dependent step in the 3-D acquisition was the baseline positioning of the probe in the initial reference planes, which were as close as possible to the coronal and sagittal planes (Fig. 3). The volume or acquisition box was adjusted to include the whole volume of interest and the same acquisition angle was used for all studies. The remaining acquisition was completely automated. The acquired data was later reconstructed into the standard views in the corresponding plane of 3-D acquisition, in line with those produced through 2-D imaging, by a research assistant (S.C.).

The 2-D study was performed immediately after the 3-D study, aiming to acquire the standard views, with additional views as required for clinical purposes. The same US machine was used with a Toshiba PVT-712BT (11MC4) 4.3–11 MHz probe.

### Image assessment

Both data sets (2-D and reconstructed 3-D) were analysed by three readers (one paediatric radiologist (S.A.) and two neuroradiologists (J.A.R. and R.M.R.), with 25 years, 7 years, and 5 years’ experience, respectively) on Apple iMac 24-inch monitors (Apple Inc., Cupertino, CA), blinded to the method of acquisition and each other. The obtained views were assessed for the visualisation of anatomical landmarks and overall quality.

Anatomical structures (Figs. 1 and 2, Table 1) were classified as present or absent as relevant for each view.

The quality of each view was graded as 0 (absent view), 1 (poor), 2 (adequate), or 3 (good), considering clarity of the image and difficulty in assessing the relevant anatomical structures as relevant for each view. Views were also classified as absent (0) when the produced image did not correspond to the standard view.

### Statistical analysis

The number of anatomical structures was determined for both the 2-D and 3-D studies, combining the results from the three different readers, using a mixed model analysis of variance (ANOVA). A similar approach was used for the quality of the views. Both the absolute values and means were used for subgroup comparison with either McNemar’s test or ANOVA, as appropriate.

A *P*-value of <0.05 was taken as statistically significant.

All statistical analysis was performed using SPSS (IBM, Armonk, NY) and Statistica (TIBCO Software, Palo Alto, CA).

## Results

A total of 40 studies were performed in 20 patients—20 2-D and 20 3-D. Twelve infants were female. The mean age at the time of scanning was 24 days (range 7–52 days). The 2-D study took a mean of 4.7 min to acquire (range 3–8 min), and the 3-D study took a mean of 3 min to acquire (range 2–5 min). The 2-D dataset comprised 28 MB, and the 3-D raw dataset 63.5 MB.

Two-way intra-class correlation coefficient (ICC) demonstrated good to excellent agreement between the three readers at 0.86 (95% confidence interval [CI] 0.83–0.88).

### Number of structures

Overall, 3-D US identified more (*n*=1,238; 80%) of the 1,540 pre-determined anatomical structures when compared with 2-D studies (*n*=1,189; 77%), reaching statistical significance (*P*<0.01).

Tables 2 and 3 show the merged results from all readers for each individual anatomical structure, grouped by each standard view for the coronal (Table 2) and sagittal (Table 3) planes.

**Table 2:** Merged results for all readers demonstrating the number of individual structures identified on 2-dimensional and 3-dimensional studies in the coronal plane, and the mean number of structures identified per view

		Individual structures identified (*n*=20)			Mean number of structures identified per view (SD)		
		2-D^a^	3-D^a^	*P-*value^b^	2-D	3-D	*P-*value^b^
Frontal lobe	R frontal lobe	14	**18**	0.22	2.48 (1.71)	3.00 (1.58)	**0.02**
	L frontal lobe	14	**18**	0.13			
	R cerebral sulci	3	**5**	0.48			
	L cerebral sulci	3	**6**	0.37			
	Interhemispheric fissure	13	**19**	**0.04**			
Frontal horns	R frontal horn	19	**20**	1.00	3.31 (1.33)	3.43 (1.21)	0.96
	L frontal horn	19	19	0.48			
	R cerebral sulci	7	**10**	0.45			
	L cerebral sulci	5	**8**	0.37			
	Interhemispheric fissure	19	**20**	1.00			
Third ventricle and Sylvian	R frontal horn	20	20	1.00	11.25 (2.68)	11.97 (1.67)	0.24
	L frontal horn	20	20	1.00			
	R deep nuclei	20	20	1.00			
	L deep nuclei	19	**20**	1.00			
	Third ventricle	12	12	0.68			
	R Sylvian fissure	18	**19**	1.00			
	L Sylvian fissure	18	18	1.00			
	R temporal lobe	18	**19**	1.00			
	L temporal lobe	18	18	1.00			
	R cerebral sulci	7	**10**	0.50			
	L cerebral sulci	8	**11**	0.45			
	Corpus callosum	19	19	0.48			
	Cavum septum	**17**	16	1.00			
	Interhemispheric fissure	20	20	1.00			
Temporal uncus and Sylvian	R Sylvian fissure	18	**19**	1.00	9.77 (2.64)	10.18 (2.67)	0.22
	L Sylvian fissure	17	17	1.00			
	R temporal uncus	19	19	1.00			
	L temporal uncus	19	19	1.00			
	Third ventricle	**15**	11	0.34			
	R transverse fissure	**18**	17	1.00			
	L transverse fissure	16	**19**	0.25			
	R cerebral peduncle	**17**	16	1.00			
	L cerebral peduncle	**17**	16	1.00			
	R cerebral sulci	10	**13**	0.45			
	L cerebral sulci	10	**15**	0.13			
	Cerebellum	19	19	1.00			
	Interhemispheric fissure	**20**	19	1.00			
Tentorium	Tentorium/cerebellum	16	**17**	1.00	5.68 (2.83)	6.02 (2.76)	0.81
	R lateral ventricle	19	19	0.48			
	L lateral ventricle	**19**	18	1.00			
	R thalamus	**17**	14	0.37			
	L thalamus	**16**	14	0.68			
	R cerebral sulci	10	**14**	0.29			
	L cerebral sulci	11	**14**	0.45			
	Interhemispheric fissure	**19**	17	0.62			
Choroid plexus	R lateral ventricle/choroid	20	20	1.00	5.97 (1.29)	6.33 (1.19)	0.10
	L lateral ventricle/choroid	**20**	19	1.00			
	R periventricular white matter	18	**20**	0.48			
	L periventricular white matter	17	**19**	0.48			
	R cerebral sulci	**14**	13	1.00			
	L cerebral sulci	13	**16**	0.25			
	Interhemispheric fissure	**20**	19	1.00			
Total		814	**857**				

^a^Bold denotes the modality which scored highest for each parameter, ^b^denotes statistical significance (*P *< 0.05)

*D* dimensional, *L* left, *R* right, *SD* standard deviation

**Table 3:** Merged results for all readers demonstrating the number of individual structures identified on 2-dimensional and 3-dimensional studies in the sagittal plane, and the mean number of structures identified per view

		Individual structures identified (*n*=20)			Mean number of structures identified per view (SD)		
		2-D^a^	3-D^a^	*P*-value	2-D	3-D	*P*-value
Midline	Genu of corpus callosum	16	**17**	1.00	5.50 (1.47)	5.20 (1.70)	0.26
	Splenium of corpus callosum	**14**	12	0.62			
	Cingulate gyrus	17	**18**	1.00			
	Surface sulci	12	12	0.62			
	Vermis	**19**	18	1.00			
	Midbrain/pons	**17**	14	0.45			
	Third ventricle	**18**	16	0.62			
Caudothalamic groove	R caudate half moon	16	**19**	0.25	4.00 (0.97)	3.81 (1.12)	0.07
	R thalamus full moon	20	**19**	1.00			
	R choroid glomus	**19**	12	**0.02**			
	R cingulate gyrus	15	15	0.62			
	R surface sulci	9	**10**	1.00			
	L caudate half moon	17	**19**	0.48			
	L thalamus full moon	**20**	19	1.00			
	L choroid glomus	**20**	14	**0.04**			
	L cingulate gyrus	15	**18**	0.37			
	L surface sulci	9	9	0.62			
Temporal	R parietal lobe	13	**17**	0.29	2.47 (1.26)	2.84 (1.10)	0.12
	R frontal lobe	8	12	0.29			
	R temporal lobe	18	**19**	1.00			
	R surface sulci	11	**13**	0.68			
	L parietal lobe	12	**15**	0.55			
	L frontal lobe	7	**13**	0.11			
	Left temporal lobe	**20**	19	1.00			
	Left surface sulci	**13**	12	1.00			
Total		375	**381**				

^a^Bold denotes the modality which scored highest for each parameter

*D* dimensional*, L* left, *R* right, *SD* standard deviation

There was a trend towards 3-D acquisition identifying more structures in the coronal plane views, with statistical significance reached for the mean number of structures in the frontal lobe view (*P*=0.02), specifically for identification of the interhemispheric fissure (*P*=0.04).

Similarly, there was a trend towards 2-D acquisition identifying more structures in the sagittal plane views, with statistical significance reached for the identification of the choroid glomus bilaterally (left *P*=0.04, right *P*=0.02).

The impact of laterality on the number of structures identified by each type of scan in the caudothalamic groove and temporal views (paired views) was further analysed (Table 4). For both views, there was a trend to identifying more structures on the left, reaching statistical significance for the caudothalamic groove view (*P*=0.01), irrespective of the mode of acquisition (*P*=0.75).

**Table 4:** Results of analysis of the impact of laterality on the mean numbers of structures identified in the caudothalamic groove and temporal views

	Mean number of structures identified per view (SD)		
	Left	Right	*P*-value^a^
Caudothalamic groove total	4.00 (0.98)	3.82 (1.12)	**0.01**
Caudothalamic groove 2-D	4.07 (0.88)	3.93 (1.06)	0.75^b^
Caudothalamic groove 3-D	3.93 (1.07)	3.70 (1.17)	
Temporal total	2.68 (1.15)	2.63 (1.24)	0.40
Temporal 2-D	2.57 (1.20)	2.37 (1.33)	0.12^b^
Temporal 3-D	2.80 (1.10)	2.88 (1.16)	

^a^Bold represents statistical significance (*P *< 0.05), ^b^Scan type and side interaction *P*-value

*D* dimensional,* SD* standard deviation

### View quality

Most studies demonstrated the pre-determined standard views, with 3-D scans attaining these more often (Table 5). Both modes of acquisition demonstrated the frontal lobe and tentorium views less frequently than the other views.

**Table 5:** Merged results for all readers presenting the proportion of views that were of adequate quality by mode of acquisition and mean view quality score

		Standard view obtained (%)		Mean view quality score (SD)		
		2-D	3-D	2-D	3-D	*P*-value^a^
Coronal views	Frontal lobe	73	83	1.37 (0.99)	1.50 (0.87)	0.23
	Frontal horns	93	97	1.63 (0.74)	1.90 (0.66)	**0.04**
	Third ventricle and Sylvian	97	100	1.82 (0.68)	2.13 (0.65)	**0.03**
	Temporal uncus and Sylvian	98	98	2.00 (0.61)	2.03 (0.71)	1.00
	Tentorium	83	88	1.87 (1.05)	1.88 (0.94)	0.94
	Choroid plexus	98	100	2.12 (0.76)	2.26 (0.72)	0.12
Sagittal views	Midline	98	100	1.97 (0.74)	1.78 (0.72)	0.30
	Caudothalamic groove	100	100	2.13 (0.63)	1.93 (0.54)	**0.02**
	Temporal	97	100	1.73 (0.63)	1.85 (0.69)	0.84

^a^Bold represents statistical significance (*P *< 0.05)

*D* dimensional,* SD *standard deviation

**Fig. 4:**
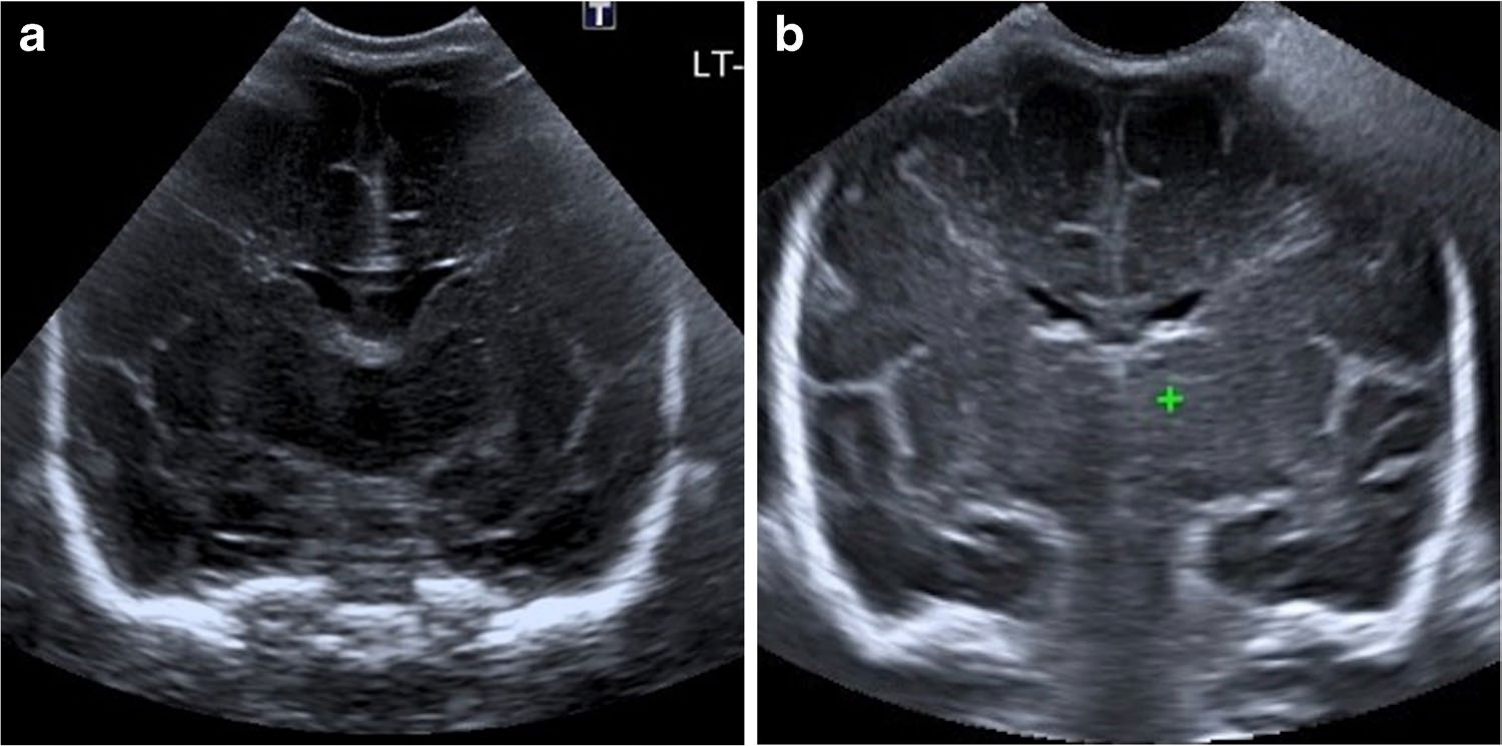
A 25-day-old girl with a normal cranial ultrasound (US). **a** Two-dimensional (D) US coronal view of the temporal uncus and Sylvian fissure. All three observers rated this view quality ‘adequate’. **b** Coronal reconstruction of the 3-D volumetric US dataset of the temporal uncus and Sylvian fissure view. Two observers rated this view quality ‘excellent’, and one ‘good’

**Fig. 5:**
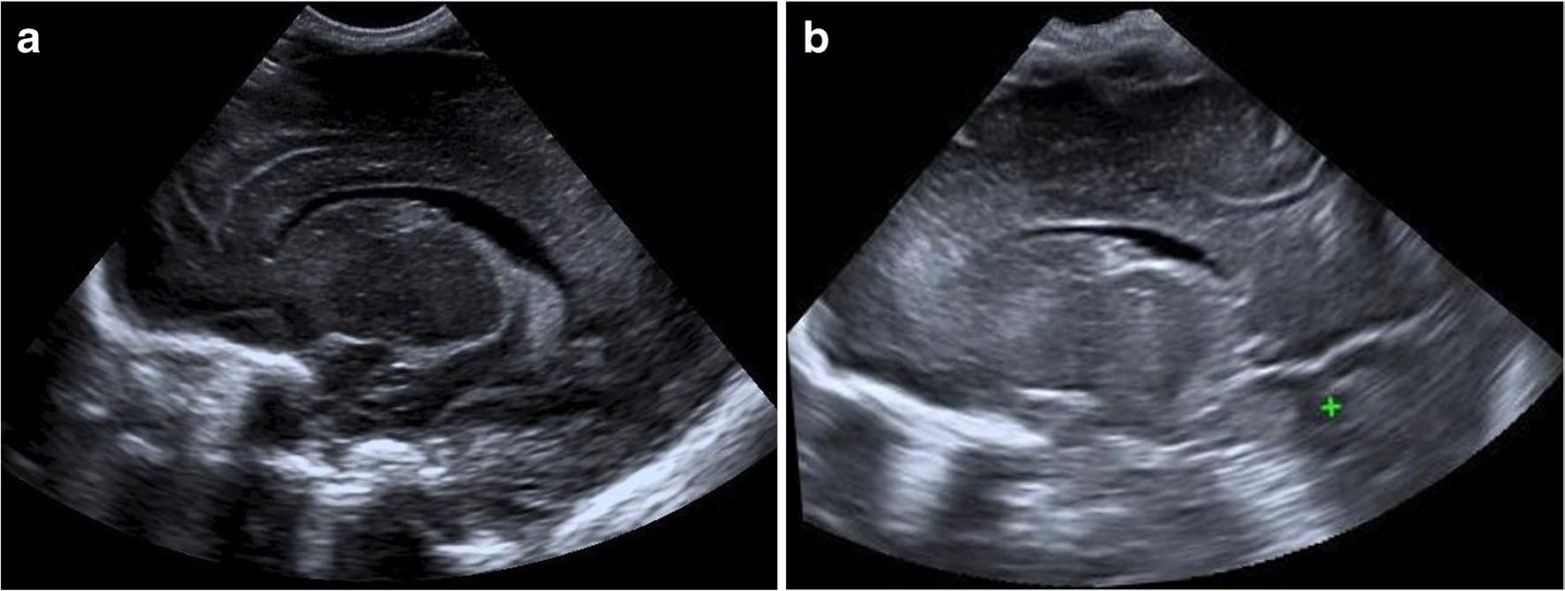
An 18-day-old girl with a normal cranial ultrasound (US). **a** Two-dimensional (D) US sagittal view of the right caudothalamic groove. One observer rated this view quality as ‘good’, and two ‘adequate’. **b** Sagittal reconstruction of the 3-D volumetric US dataset of the right caudothalamic groove view. Note the pivot point used for 3-D manipulation and reconstruction (*caliper*). The image also demonstrates the smaller near field view of the 3-D acquisition. One observer rated this view quality as ‘good’, one ‘adequate’, and one ‘poor’

The mean quality of each standard view for 2-D and 3-D acquisitions is also presented in Table 5. The 3-D studies produced higher quality images for all coronal plane views, with an example demonstrated in Fig. 4 reaching statistical significance for the frontal horns (*P*=0.04) and third ventricle and Sylvian (*P*=0.03) views. The 2-D studies were of higher quality for the sagittal plane views, reaching statistical significance for the caudothalamic groove view (*P*=0.02) (Fig. 5).

The impact of laterality on view quality for each type of scan in the caudothalamic groove and temporal views was further analysed (Table 6). Irrespective of the type of scan, there was a trend to superior quality on the left, although not statistically significant.

**Table 6:** Results of analysis of the impact of laterality on the mean view quality of the caudothalamic groove and temporal views

	Mean view quality score (SD)		
	Left	Right	*P*-value
Caudothalamic groove total	2.09 (0.60)	1.96 (0.59)	0.05
Caudothalamic groove 2-D	2.18 (0.65)	2.07 (0.61)	0.87^a^
Caudothalamic groove 3-D	2.00 (0.52)	1.85 (0.55)	
Temporal total	1.80 (0.62)	1.78 (0.72)	0.25
Temporal 2-D	1.75 (0.54)	1.70 (0.72)	0.54^a^
Temporal 3-D	1.85 (0.68)	1.85 (0.71)	

^a^Scan type and side interaction *P*-value

*D* dimensional, *SD* standard deviation

### Presence of pathology

Overall, the presence of pathology reduced the number of anatomical landmarks identified in all views, affecting both 2-D and 3-D studies (Table 7). When the type of study was not considered, pathology significantly reduced the number of structures identified in multiple views. However, the interaction between pathology and type of study annulled this significance for most views, except for the temporal view (*P*=0.02), where post hoc analysis revealed that pathology affected the performance of both types of US, although with greater effect on 3-D acquisitions: 2-D (*P*=0.02) and 3-D (*P*<0.01).

**Table 7:** The impact of the presence of pathology and type of scan on the mean number of structures identified per view

		Pathology absent	Pathology present	*P*-value^a^	Pathology absent		Pathology present		*P*-value ^a, b^
		All studies			2-D	3-D	2-D	3-D	
Coronal views	Frontal lobe	2.78 (1.77)	2.63 (1.33)	0.79	2.58 (1.84)	2.98 (1.69)	2.20 (1.26)	3.07 (1.28)	0.39
	Frontal horns	3.46 (1.33)	3.13 (1.07)	0.33	3.33 (1.48)	3.58 (1.16)	3.27 (0.80)	3.00 (1.30)	0.28
	Third ventricle and Sylvian	11.96(1.67)	10.6 (2.05)	0.07	11.47 (2.84)	12.44 (1.20)	10.60 (2.10)	10.53 (2.07)	0.18
	Temporal uncus and Sylvian	10.7 (1.61)	7.83 (3.82)	**0.01**	10.47 (1.66)	10.91 (1.55)	7.67 (3.81)	8.00 (3.96)	0.86
	Tentorium	6.48 (2.50)	3.97 (2.80)	**0.01**	6.20 (2.63)	6.76 (2.35)	4.13 (2.90)	3.80 (2.78)	0.34
	Choroid plexus	6.51 (0.84)	5.07 (1.62)	0.01	6.27 (1.03)	6.76 (0.48)	5.07 (1.58)	5.07 (1.70)	0.10
Sagittal views	Midline	5.61 (1.35)	4.57 (1.98)	0.10	5.78 (1.18)	5.44 (1.49)	4.67 (1.91)	4.47 (2.10)	0.78
	Caudothalamic groove	4.21 (0.85)	3.00 (1.18)	**0.01**	4.3 (0.76)	4.12 (0.85)	3.10 (0.99)	2.90 (1.35)	0.91
	Temporal	2.92 (1.08)	1.85 (1.18)	**0.01**	2.66 (1.23)	3.18 (0.83)	1.90 (1.21)	1.80 (1.16)	**0.02**

^a^Bold represents statistical significance (P<0.05), ^b^Scan type and presence of pathology interaction *P*-value

*D* dimensional

**Fig. 6:**
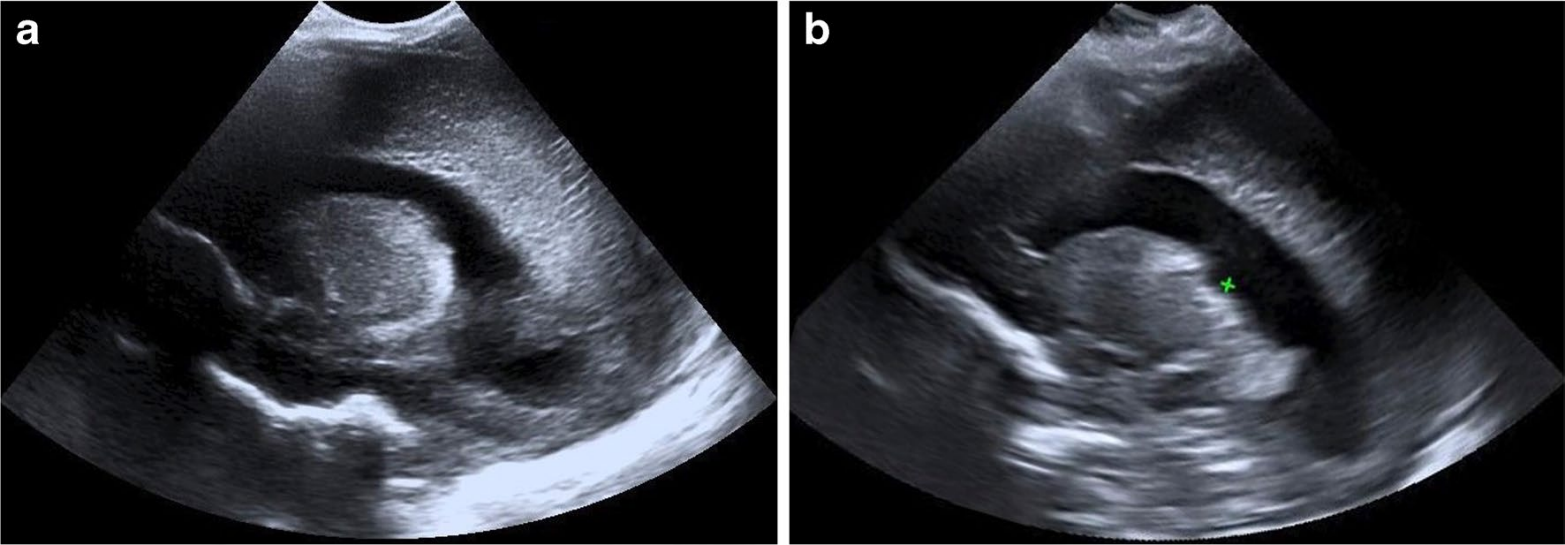
A 14-day-old girl with hydrocephalus. **a** Two-dimensional (D) ultrasound (US) sagittal view of the right caudothalamic groove demonstrates hydrocephalus and increased periventricular echogenicity. One observer rated this view quality ‘poor’, and two ‘adequate’. **b** Sagittal reconstruction of the 3-D volumetric US dataset of the right caudothalamic groove view demonstrates the smaller near field view of the 3-D acquisition. Note the pivot point used for 3-D manipulation and reconstruction (*caliper*). One observer rated this view quality ‘adequate’, and two ‘poor’

Similarly, the presence of pathology reduced quality in all views irrespective of the type of study, as seen in Fig. 6. This was significant for multiple views when the type of study was not considered (Table 8). The combined effect of pathology and the type of study was only significant in the temporal view (*P*<0.01). Post hoc analysis revealed this resulted from a significant effect on 3-D performance (*P*<0.01).

**Table 8:** The impact of the presence of pathology and type of scan on the mean view quality

		Pathology absent	Pathology present	*P*-value^a^	Pathology absent		Pathology present		*P*-value^a,b^
		All studies			2-D	3-D	2-D	3-D	
Coronal views	Frontal lobe	1.49 (0.99)	1.27 (0.74)	0.47	1.44 (1.06)	1.53 (0.92)	1.13 (0.74)	1.40 (0.74)	0.55
	Frontal horns	1.85 (0.71)	1.5 (0.63)	0.11	1.71 (0.79)	2.00 (0.60)	1.40 (0.51)	1.60 (0.74)	0.71
	Third ventricle and Sylvian	2.09 (0.65)	1.63 (0.67)	**0.04**	1.89 (0.68)	2.29 (0.55)	1.60 (0.63)	1.67 (0.72)	0.13
	Temporal uncus and Sylvian	2.19 (0.56)	1.5 (0.61)	**0.01**	2.16 (0.52)	2.22 (0.60)	1.53 (0.64)	1.47 (0.74)	0.52
	Tentorium	2.08 (0.95)	1.27 (0.87)	**0.01**	2.07 (1.00)	2.09 (0.90)	1.27 (0.96)	1.27 (0.80)	0.94
	Choroid plexus	2.33 (0.64)	1.77 (0.63)	**0.02**	2.27 (0.72)	2.40 (0.54)	1.67 (0.72)	1.87 (0.52)	0.75
Sagittal views	Midline	1.92 (0.75)	1.73 (0.64)	0.49	2.04 (0.73)	1.80 (0.76)	1.73 (0.70)	1.73 (0.59)	0.30
	Caudothalamic groove	2.13 (0.55)	1.7 (0.60)	**0.02**	2.26 (0.57)	2.01 (0.51)	1.73 (0.64)	1.67 (0.55)	0.18
	Temporal	1.91 (0.67)	1.42 (0.50)	**0.01**	1.78 (0.67)	2.04 (0.65)	1.57 (0.50)	1.27 (0.45)	** <0.01**

^a^Bold denotes statistical significance (*P *< 0.05), ^b^Scan type and presence of pathology interaction *P*-value

*D* dimensional

## Discussion

Previous studies demonstrated similar performance between 2-D and 3-D US in identifying intracranial pathology and depicting it with diagnostic quality in the neonatal population. In addition, 3-D US has shown reduced acquisition times which is replicated in our study [9].

Diagnostic confidence has also been demonstrated to be higher for 3-D scans when images are interpreted by a reader other than the operator because the whole volume of interest is acquired irrespective of the operator’s proficiency [9].

Although the main purpose of any diagnostic tool is to correctly identify pathology, it is first necessary to ensure that the imaging modality being used can depict all the relevant anatomy. To date, it had not been demonstrated that 3-D US could perform at least equally to 2-D in this respect, with the ability to obtain the views considered necessary to comprise a neonatal cranial study that meets the internationally recognised quality criteria [18].

Neonatal cranial 3-D ultrasound image acquisition by an onsite operator with little training can provide high-quality and accurate anatomic digital images for interpretation at a distant site by an expert. This lack of location dependence is a useful attribute in all countries, with a current and projected worsening shortage of paediatric radiologists in high- and upper–middle-income countries which may result in residents or radiologists with limited paediatric neuroradiology experience performing such studies, as well as the significant role ultrasound is playing as an affordable mobile imaging modality in lower–middle and low-income countries.

The increased data required for storage on picture archiving and communication systems should be considered a potential implication if most neonatal transcranial US was instead acquired as a 3-D study in the future. However, this should be weighed against the other potential benefits of 3-D US considered in this discussion. For our study, it was not a consideration, as the additional data was held in research storage.

The current study demonstrates that semi-automated 3-D cranial US outperforms 2-D US in demonstrating anatomical structures in all standard coronal views, significantly in the frontal lobe view.

On sagittal views, although the mean number of structures per view is superior for the 2-D studies, this does not reach significance for any view and, overall, 3-D US identifies more structures.

The pre-determined standard views are obtained more often by the 3-D studies, except for the temporal uncus and Sylvian view, where both type of US perform equally. This likely reflects the fact that the 3-D US does in fact acquire the whole volume of interest and can therefore be used to successfully reconstruct images in any plane at a later stage.

Regarding the quality of each standard view, 3-D also outperformed 2-D in all coronal and temporal views, while 2-D performed better in the remaining sagittal views. However, 3-D is only significantly superior for the frontal horns and third ventricle and Sylvian views, and 2-D for the caudothalamic groove view.

These differences in performance, with 3-D being overall slightly superior in the coronal plane and 2-D in the sagittal plane, are likely due to the different modes of acquisition. During the 2-D acquisition, the operator tilts the probe to obtain a better parasagittal view whilst the 3-D probe location is fixed for the 3-D acquisition. Additionally, as 2-D acquisition is operator dependent, the operator is permitted to adjust settings freely during the 2-D acquisition, according to their expert judgement at the time, whilst during 3-D acquisition, no adjustment of the ultrasonographic parameters occurs.

This also likely explains why the 3-D acquisition seems to be most affected by the presence of pathology in the most extreme lateral sagittal view, the temporal view, as during the 2-D acquisition the operator is free to adjust their view as necessary to best depict the underlying anatomy. The views from the 3-D acquisition were reconstructed by a research assistant and the readers did not have access to the whole acquired volume due to the intended blinded nature of the study, which would not be the case in clinical practice. In practice, the interpreting radiologist or physician would be able to reconstruct in any plane as required.

For paired views, the tendency to better image quality as well as more anatomical structures identified on the left is also likely a result of the above factors, as well as patient positioning and handedness of the operator. This should be given further consideration in the proposed larger scale study.

The main strength of this study is that all the 2-D US studies were performed by the same experienced operator and the single non-automated step of the 3-D acquisition was also performed by that same operator in all patients, reducing potential confounding factors. Additionally, most patients in this study did not have intracranial pathology and therefore the performance of both types of acquisition in demonstrating anatomical structures could be assessed without significant confounding factors.

This study has limitations, one of the main ones being the restricted access the readers had to the entire acquired 3-D volume, as they could only assess reconstructed images. Therefore, the real-life performance of 3-D US is likely to be superior to that demonstrated in this study. The whole acquired volume should be included in a larger scale study, as well as a 2-D sweep, to allow access to as much data as possible by the reader for each type of study and better reproduce real life clinical practice.

A further limitation is that although efforts were made to blind the readers to the nature of the US acquisition, there is a clear difference between the images of a 2-D acquired view and a view reconstructed from a 3-D volume, with a narrower near field in the 3-D view, demonstrated in Figs. 4, 5, and 6. Therefore, the readers were able to infer the mode of acquisition.

Additionally, the patients included in the study were a mix of term and pre-term infants. We did not collect age-related data. This may have an impact on the findings of a neonatal US, and should be considered in any future study.

The overall number of patients included was small but adequate for a proof-of-concept study, and it has been demonstrated that a similar study with a larger number of patients is feasible at our institution.

The group with pathology was smaller, and although some of the findings showed statistical significance in this subgroup, these should be seen as trends. They are however relevant findings that should inform the design of further studies and be investigated with a larger number of patients.

## Conclusion

Semi-automated 3-D acquired neonatal cranial US performs similarly to 2-D US performed by an experienced operator, both at detecting anatomical structures and in producing good quality images. This could enable clinicians to acquire quality cranial US using a semi-automated study in unwell neonates at remote sites when experienced operators are unavailable. Images could then be interpreted remotely by expert radiologists, avoiding long transfers of these frequently unwell patients.

The authors aim to extend the current proof-of-concept study to a larger number of patients. The successful completion of this proof-of-concept study has permitted a power calculation to be performed based on a mixed model ANOVA for the detection of differences between 2-D and 3-D quality. This suggests that a sample size of 50 would be sufficient for a definitive future study. 

## Data Availability

The data that support the findings of this study are not openly available due to reasons of sensitivity and are available from the corresponding author upon reasonable request. Data are located in controlled access data storage at St Michael’s Hospital, Bristol.
